# Mindful awareness and its association with cumulative adversity, autonomic reactivity, and current functioning

**DOI:** 10.3389/fpsyg.2025.1630101

**Published:** 2025-07-23

**Authors:** Audrey N. Dana, Jenna L. Pennella, Donnalea Van Vleet Goelz, Britney N. Duner, Allison B. Ventura, Sara M. Giraldo, Steven P. Cuffe, Stephen W. Porges, Lourdes P. Dale

**Affiliations:** ^1^Department of Psychiatry, College of Medicine-Jacksonville, University of Florida, Jacksonville, FL, United States; ^2^Department of Psychology, Florida State University, Tallahassee, FL, United States; ^3^Department of Psychology, University of North Florida, Jacksonville, FL, United States; ^4^Traumatic Stress Research Consortium, Kinsey Institute, Indiana University, Bloomington, IN, United States

**Keywords:** mindful awareness, adversity, autonomic reactivity, dysfunctional coping strategies, emotional distress, PTSD

## Abstract

**Background:**

Prior adversity may retune autonomic regulation and impede the ability to access a calm physiological state that would support mindful awareness, which is the facet of mindfulness focused on being present and accepting of the current moment. We investigated (1) how mindful awareness relates to cumulative adversity, autonomic reactivity, and current functioning; and (2) whether mindful awareness ameliorates the negative impact of cumulative adversity and autonomic reactivity on current functioning.

**Methods:**

Participants (*N* = 1,542; ages 18–88) reported living in the United States and experiencing at least one adversity. They completed online measures assessing their mindful awareness, cumulative adversity, autonomic reactivity, dysfunctional coping strategies, emotional distress, and PTSD symptoms.

**Results:**

Regression analyses revealed that the strongest predictor of greater mindful awareness was lower autonomic reactivity, which minimized the impact of cumulative adversity. Mediational analyses suggested that autonomic reactivity may mediate the relationship between cumulative adversity and mindful awareness. Regression analysis suggested higher autonomic reactivity remained the strongest predictor of poorer current functioning.

**Conclusion:**

Our research suggests that individuals with adversity histories may need to address both their mindful awareness and autonomic dysregulation to improve their current functioning.

## 1 Introduction

Mindfulness refers to being present and attending to thoughts, behaviors, bodily sensations, and emotions in a non-reactive and non-judgmental way (Kabat-Zinn, 2015). Available research suggests that mindfulness may help individuals in acknowledging and controlling their thoughts, feelings, and emotions (Hilt et al., 2023; Shapiro et al., 2006), being accepting and less self-blaming (Finkelstein-Fox et al., 2019), and in feeling gratitude toward the positive aspects of their lives (Swickert et al., 2018). By encouraging individuals to work toward more adaptive ways of thinking, mindfulness helps individuals to let go of negative thoughts (Frewen et al., 2008), alleviate stress (Fjorback et al., 2011), and decrease the use of maladaptive coping strategies (Charoensukmongkol, 2013), such as rumination and disengagement (Hilt et al., 2023; Cousin and Crane, 2016). Mindful awareness, which is the facet of mindfulness focused on being present and accepting of the current moment (Brown and Ryan, 2003), may lead to improved emotional functioning (Swickert et al., 2018; Brown and Ryan, 2003).

A group that may have difficulty practicing mindful awareness is individuals who report being impacted by their prior adversity/trauma history because their experiences not only impact the cognitive perception of the danger, but also the body’s neuroception, or ability to independent of consciousness accurately perceive safety and danger, as explained in polyvagal theory (Porges, 1995, 2007, 2011; 2023). If danger is detected, the sympathetic nervous system signals the body to disengage through mobilization (fight or flight), which may lead to chronic anxiety and/or irritability, or through immobilization (freeze), which may lead to death feigning, syncope, dissociation, withdrawal, loss of purpose, social isolation, and depression. These stress reactions are associated with increased autonomic reactivity, which may lead to a focus on self-protection, rather than cultivating bodily awareness (or interoception) to sharpen the communication within and beyond ourselves (Van Vleet Goelz, 2025). Thus, these individuals may not notice physical tension/discomfort until it becomes severe, and potentially debilitating. In addition, they may experience increased autonomic reactivity, which may lead to increased use of maladaptive coping strategies and psychiatric disorders (Anda et al., 2008; McLaughlin et al., 2012; O’Connor et al., 2002; Street et al., 2005). Prior research (Brunoni et al., 2013; Cohen et al., 1998; Kemp et al., 2012; Kolacz et al., 2025) suggests an association between autonomic dysregulation and mental health disorders (i.e., depression, anxiety, and PTSD).

In the current study, we investigated self-reported mindful awareness and its relation to the risk factors of cumulative adversity and increased autonomic reactivity. Rather than focusing on the occurrence of an adverse or traumatic event, we focused on the perceived impact of the experience because individuals more impacted by their adversity history may experience difficulties with autonomic regulation (Dale et al., 2022; Kolacz et al., 2020). We used a self-report measure of autonomic reactivity, which prior research found to be clinically useful, as increased levels of autonomic reactivity were associated with higher resting heart rate, lower parasympathetic activity, and less parasympathetic/sympathetic flexibility in response to challenges (Kolacz et al., 2023). Contrary to prior research that focused on mindfulness as an outcome variable (Dale et al., 2022), we explored how mindful awareness may relate to aspects of current functioning (i.e., dysfunctional coping strategies, emotional distress, and PTSD symptoms) that may be negatively impacted by adversity and positively impacted by mindful awareness.

Thus, the current study expands on prior research by examining the factors that impact and are associated with mindful awareness. Specifically, we explored how cumulative adversity, autonomic reactivity, and demographic factors (i.e., age, gender, race, education level, and yearly household income) relate to mindful awareness, and hypothesized that:

• Cumulative adversity and autonomic reactivity would be associated with decreased mindful awareness, even when controlling for demographic factors.
• Autonomic reactivity would mediate the relationship between cumulative adversity and mindful awareness.

Given that prior research supports many benefits of mindful awareness, including improvements in cognitive and emotional functioning, decreased stress, improved sustained attention, and increased use of adaptive coping strategies (Hilt et al., 2023; Fjorback et al., 2011; Schmalzl et al., 2018; Swickert et al., 2018), we hypothesized the following:

• Mindful awareness would be associated with decreased dysfunctional coping strategies, emotional distress, and PTSD symptoms.
• Mindful awareness would be associated with a reduction in the negative impact of cumulative adversity and autonomic reactivity on dysfunctional coping behaviors, emotional distress, and PTSD symptoms.

## 2 Materials and methods

### 2.1 Recruitment procedure

Participants aged 18 years or older were recruited via social media posts on Reddit, Twitter, Facebook, Instagram, and email lists, and these individuals were not paid for their participation. Additional participants were recruited through Qualtrics Panels in order to increase the percentage of male, low income, and non-Caucasian participants, and were paid according to their compensation plan (e.g., cash, airline miles). This manuscript only focuses on the participants who reported experiencing being impacted by at least one lifetime adversity and living within the United States.

### 2.2 Measures

The survey collected demographic data, such as gender, racial identity, age, and education and income level. Below is a description of the constructs and measures, and the analyses assessing internal consistency of the measures via Cronbach alpha (α).

#### 2.2.1 Mindful awareness

We used the Mindful Attention Awareness Scale (Brown and Ryan, 2003; Carlson and Brown, 2005), which assesses awareness of and attention to what is taking place in a present moment. This 15-item measure asks participants to indicate frequency of mindfulness events occur via a six-point Likert scale (1 = *almost always* to *6* = *almost never*). Higher means scores suggest higher levels of dispositional mindfulness. This scale has been found to be internally consistent in the current sample (α = 0.90).

#### 2.2.2 Risk factors

Cumulative adversity was assessed through a preliminary version of the Adverse and Traumatic Experiences Scale (Dale et al., 2020), which asks participants to indicate how impacted they were using a five-point Likert scale (0 = *event did not occur* to 3 = *big impact on my life*) by various adversities. Items focused on adverse childhood environment (six items), caregiver abuse (three items), non-caregiver interpersonal adversity (two items), life-threatening situations (four items), and physical health problems (six items) were combined to form a cumulative adversity impact score (α = 0.84).

Autonomic reactivity was assessed via the Body Perception Questionnaire Short Form (Kolacz et al., 2018; Porges, 1993), which assesses experiences of reactivity in organs and tissues regulated by the autonomic nervous system. This 20-item questionnaire assesses frequency of specific bodily sensations via a five-point Likert scale (1 = *never* to 5 = *always*). Higher scores indicate increased autonomic reactivity.

#### 2.2.3 Current functioning

Dysfunctional coping strategies was assessed via the 21-item Regulations of Emotions Questionnaire-2 (Phillips and Power, 2007), which assesses frequency of engaging in strategies to recognize, monitor, evaluate, and modify emotional reactions via a five-point Likert scale (0 = *never* to 4 = *always*). We combined items from the subscales assessing internal and external dysfunctional coping strategies scales (e.g., rumination, negative social comparison, verbal/physical assault, and bullying) to derive the internally consistent (α = 0.87) dysfunctional coping strategies score.

Emotional distress was assessed via a 12-item measure that asks frequency via a five-point Likert scale (0 = *not at all* to 4 = *extremely*) of signs of distress symptomatology listed in the Center for Disease Control Website (e.g., anger/fear, sadness, bothered by things that did not bother them before, everything feels like an effort, feelings of disbelief, and increased substance use). Item scores were combined to derive an internally-consistent total score (α = 0.92), with higher scores indicating greater emotional distress.

PTSD symptoms were assessed via the 17-item PTSD Checklist Civilian Version (Weathers et al., 1991), which assess level of distress within the last month via a five-point Likert scale (0 = *not all* to 4 = *extremely*). This measure has been found to be internally consistent with the current sample (α = 0.95).

### 2.3 Data analysis

Data was transferred from Qualtrics to IBM SPSS Statistics for Windows, Version 29.0.2.0 Armonk, NY: IBM Corp. Data analyses included calculating the internal consistency of the measures via Cronbach alpha analyses. Descriptive statistics determined demographic characteristics (e.g., gender, race, education level, and yearly household income), history of prior adversities, and psychiatric history. Additional analysis determined the means and standard deviations of the variables in the study.

Pearson correlational analyses determined which factors were statistically related to mindful awareness. Because of potential covariation, hierarchical stepwise linear regression analyses explored the contributions of cumulative adversity (entered in block 1) and autonomic reactivity (entered in block 2) in predicting mindful awareness. Block 3 included the demographic variables previously found to be related to mindful awareness to determine if they impacted the significance of the already identified significant predictors.

**FIGURE 1:**
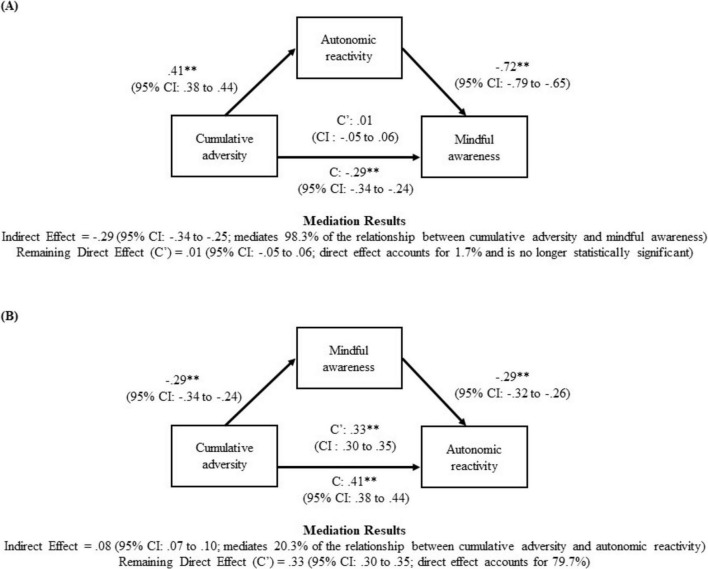
Autonomic reactivity mediates the relationship between cumulative adversity and mindful awareness. It depicts the most statistically likely relationship between cumulative adversity, autonomic reactivity, and mindful awareness. The hypothesized mediation model used a bias-corrected 95% confidence intervals (*n* = 10,000), bootstrapping approach to assess the significance of the indirect effects. The results suggest that autonomic reactivity mediates the relationship between cumulative adversity and mindful awareness. **(A)** Indirect Effect = –0.29 (95% CI: –0.34 to –0.25; mediates 98.3% of the relationship between cumulative adversity and mindful awareness) Remaining Direct Effect (C’) = 0.01 (95% CI: –0.05 to.06; direct effect accounts for 1.7% and is no longer statistically significant). **(B)** Indirect Effect = 0.08 (95% CI: 0.07 to 0.10; mediates 20.3% of the relationship between cumulative adversity and autonomic reactivity) Remaining Direct Effect (C’) = 0.33 (95% CI: 0.30 to 0.35; direct effect accounts for 79.7%). **p < 0.01.

Mediational analyses via “PROCESS” macro model 4 (Hayes, 2013) investigated whether autonomic reactivity mediated the relationship between cumulative adversity and mindful awareness (see Figure 1A). The alternative hypothesis that mindful awareness mediated the relationship between the cumulative adversity and autonomic reactivity (Figure 1B) was also examined to determine the most statistically likely ordering of the variables. The hypothesized mediation model (see Figure 1) used a bias-corrected 95% confidence intervals (*n* = 10,000), bootstrapping approach to assess the significance of the indirect effects. For these analyses, significant effects are supported by the absence of zero within the confidence intervals.

Next, hierarchical stepwise linear regression analyses determined the contributions of cumulative adversity (entered in block 1), autonomic reactivity (entered in block 2), and mindful awareness (entered in block 3) on the current functioning variables. The demographic variables were entered in block 4 to determine if they impacted the findings.

## 3 Results

### 3.1 Description of participants

Table 1 describes the sample (*N* = 1,542), which were individuals living in the United States that varied in age from 18 to 88 years old (*M* = 48.11, *SD* = 15.71). Although they differed with regard to gender, race/ethnicity, educational level, and yearly household income, they were predominantly individuals who reported being female, Caucasian, and having a graduate level education. Even though all participants reported being impacted by at least one prior adversity, they varied in their report of how impacted they were by specific adversities. As reported in Table 1, most individuals reported being impacted by their adverse childhood environment (85.5%) and physical health problems (71.8%).

**TABLE 1:** Demographic factors and psychiatric history.

Characteristic	*N*	%	Characteristic	*N*	%
**Gender**			**Race**		
Female	1,043	67.9	Asian	64	4.2
Male	471	30.7	Black	96	6.3
Other	21	1.4	Caucasian	1,197	78.1
**Education level**			Hispanic	75	4.9
≤ High school	213	13.8	**Prior adversities**		
College/University	541	35.1	Adverse childhood environment	1,319	85.5
Graduate school	788	51.1	Caregiver abuse	702	45.5
**Yearly household income**			Non-caregiver interpersonal adversity	623	40.4
≤ $20,000	175	11.4	Life-threatening situations	821	53.2
$20,001–$40,000	256	16.7	Physical health problems	1,107	71.8
$40,001–$60,000	218	14.3	**Above PTSD clinical cutoff**	406	26.3
$60,001–$80,000	246	16.1			
$80,001–$100,000	177	11.6			
$100,001–$200,000	342	22.4			
≥ $200,001	115	7.5			

As reported in Table 2, Pearson correlational analyses found that participants varied in their report of the extent of their mindful awareness. Demographic differences in the report of mindful awareness emerged. Greater mindful awareness was reported by participants identified as male (*r* = 0.12, *p* < 0.001) and/or Black/African American (*r* = 0.09, *p* < 0.001), and reported being older (*r* = 0.29, *p* < 0.001) and/or having less education (*r* = −0.09, *p* < 0.001).

**TABLE 2:** Correlates of mindful awareness: results of Pearson correlational analyses.

	*M* (*SD*)	1	2	3	4	5
1. Mindful awareness	4.06 (0.90)	–				
2. Cumulative adversity	17.48 (12.88)	−0.28^***^	–			
3. Autonomic reactivity	48.10 (9.74)	−0.52^***^	0.54^***^	–		
4. Dysfunctional coping strategies	1.76 (1.31)	−0.51^***^	0.43^***^	0.57^***^	–	
5. Emotional distress	25.92 (10.31)	−0.49^***^	0.38^***^	0.55^***^	0.59^***^	–
6. PTSD symptoms	35.40 (15.58)	−0.53^***^	0.45^***^	0.59^***^	0.63^***^	0.85^***^

***p < 0.001.

### 3.2 Association between cumulative adversity, autonomic reactivity, and mindful awareness

As reported in Table 2, Pearson correlational analyses found that individuals more impacted by their cumulative adversity and those with increased autonomic reactivity reported less mindful awareness. As shown in Table 3, hierarchical stepwise linear regression analyses predicting mindful awareness indicated that cumulative adversity alone accounted for 7.6% of the variability in mindful awareness. In step 2, the inclusion of autonomic reactivity reduced the significance of cumulative adversity in predicting mindful awareness and significantly increased the percent accounted for to 26.8%. Step 3 indicated that older age, lower education, male gender, and Black, Asian, and Hispanic race/ethnicity were associated with an increase in mindful awareness. The inclusion of these demographic variables did not significantly diminish the impact of autonomic reactivity, as this remained the strongest predictor of mindful awareness (standardized beta coefficient of −0.45). The combination of all variables accounted for 34.7% of the variability in mindful awareness.

**TABLE 3:** Hierarchical stepwise linear regression predicting mindful awareness.

Predictors	Step 1 β	Step 2 β	Step 3 β	Step 4 β	Step 5 β	Step 6 β
Cumulative adversity	−0.28^***^	0.00	−0.03	−0.05	−0.04	−0.05
Autonomic reactivity		−0.52^***^	−0.46^***^	−0.45^***^	−0.45^***^	−0.45^***^
Age			0.19^***^	0.22^***^	0.23^***^	0.23^***^
Black/African American				0.15^***^	0.13^***^	0.12^***^
Education level					−0.11^***^	−0.09^***^
Male						0.07^***^
*R*^2^ change	0.077^***^	0.190^***^	0.036^***^	0.022^***^	0.011^***^	0.005^***^
*R* ^2^	0.077	0.267	0.302	0.325	0.336	0.341
*F*	122.46^***^	266.31^***^	211.25^***^	175.70^***^	147.88^***^	125.97^***^

***p < 0.001.

Because autonomic reactivity diminished the impact of cumulative adversity on mindful awareness, mediational analyses explored whether autonomic reactivity may mediate the relationship between cumulative adversity and mindful awareness (Figure 1). Figure 1A presents the indirect, direct, and total effects of this analysis, and reports that autonomic reactivity mediated 98.3% of the relationship between cumulative adversity and mindful awareness. Conversely, the results presented in Figure 1B, which examined the alternative mediational relationship, suggested that mindful awareness is less likely to mediate the relationship between the cumulative adversity and autonomic reactivity, as mindful awareness only mediated 20.3% of the relationship. Thus, the mediation analyses suggested that the more statistically likely relationship is that cumulative adversity may be associated with increased autonomic reactivity, which may be linked with less mindful awareness.

### 3.3 Mindful awareness and current functioning

As reported in Table 2, individuals that reported being more mindful reported engaging in less dysfunctional coping strategies, and experiencing less emotional distress and PTSD symptoms. Table 4 displays the results of the hierarchical stepwise line regression analyses predicting each of the current functioning variables (i.e., dysfunctional coping strategies, emotional distress, and PTSD symptoms). Cumulative adversity was associated with an increase in dysfunctional coping strategies, emotional distress, and PTSD symptoms. The inclusion of autonomic reactivity in step 2 significantly reduced but did not eliminate the impact of cumulative adversity for all the current functioning variables. When mindful awareness was included in step 3, it was associated with less current functioning difficulties and ameliorated the impact of autonomic reactivity. The demographic factors did not impact the strengths of the associations, and thus they are not presented below and in Table 4. The standardized beta coefficients from the final model indicated that autonomic reactivity remained the most significant predictor of dysfunctional coping strategies, emotional distress, and PTSD symptoms, and that the combination of all predictors accounted for 42.3% of the variability in dysfunctional coping strategies, 38.2% of the variability in emotional distress, and 45.6% of the variability in PTSD symptoms.

**TABLE 4:** Hierarchical stepwise linear regression predicting current functioning.

Current functioning	Predictors	Step 1 β	Step 2 β	Step 3 β
**Dysfunctional coping strategies** *F*(3, 1,449) = 353.57***	Cumulative adversity	0.44^***^	0.18^***^	0.18^***^
	Autonomic reactivity		0.48^***^	0.33^***^
	Mindful awareness			−0.30^***^
	*R*^2^ change	0.194^***^	0.164^***^	0.065^***^
	*R* ^2^	0.194	0.358	0.423
**Emotional distress** *F*(3, 1,449) = 298.59***	Cumulative adversity	0.39^***^	0.13^***^	0.13^***^
	Autonomic reactivity		0.49^***^	0.33^***^
	Mindful awareness			−0.29^***^
	*R*^2^ change	0.153^***^	0.167^***^	0.062^***^
	*R* ^2^	0.153	0.320	0.382
**PTSD symptoms** *F*(3, 1,449) = 405.25***	Cumulative adversity	0.47^***^	0.20^***^	0.21^***^
	Autonomic reactivity		0.48^***^	0.32^***^
	Mindful awareness			−0.32^***^
	*R*^2^ change	0.217^***^	0.166^***^	0.074^***^
	*R* ^2^	0.217	0.382	0.456

***p < 0.001. Inclusion of the demographic factors in step 4 did not impact the findings.

## 4 Discussion

### 4.1 Summary of findings

We explored with a large sample (*N* = 1,542) of individuals, who reported experiencing at least one adversity, their level of mindful awareness and whether it relates to their cumulative adversity, autonomic reactivity, and current functioning. As hypothesized, we found that both cumulative adversity and increased autonomic reactivity were associated with decreased levels of mindful awareness, a finding that is consistent with prior research (Bullis et al., 2014; Scavone et al., 2020) suggesting that autonomic dysregulation is associated with decreased levels of mindful awareness. However, statistical analysis suggested that autonomic reactivity was the stronger predictor, and that it may mediate the relationship between cumulative adversity and mindful awareness. Thus, our results suggest that individuals with adversity histories who report increased levels of autonomic reactivity may have more difficultly staying fully present and non-reactive, a finding that is consistent with the belief that prior adversity may results in a dysregulated nervous system (Porges, 1995, 2007, 2009).

Consistent with our hypothesis, we found an association between mindful awareness and the current functioning variables of dysfunctional coping strategies, emotional distress, and PTSD symptoms. Our finding of less dysfunctional coping strategies in individuals with higher levels of mindful awareness is consistent with research suggesting that mindful awareness may lead to more adaptive ways of thinking and behaving (Charoensukmongkol, 2013). Additionally, our finding that mindful awareness was linked to less emotional distress and PTSD symptoms is also consistent with research suggesting that mindful awareness is associated with improved emotional functioning (Swickert et al., 2018) and decreased PTSD symptoms (Harper et al., 2022). Thus, mindfulness-based practices may be useful in decreasing the autonomic reactivity associated with prior adversity (Harper et al., 2022), which is important as autonomic reactivity statistically emerged as a stronger predictor of current functioning than mindful awareness.

Our combined results suggest that mindful awareness may help to diminish the negative impact of cumulative adversity and increased autonomic reactivity. Thus, individuals with adversity histories may benefit from interventions that include mindful awareness, which may allow them to reappraise their adverse experiences and develop greater cognitive flexibility and perspective on their experiences (Garland et al., 2015). This may decrease the need to engage in dysfunctional coping strategies and lead to improved emotion regulation abilities.

Because of our large sample size, we were able to investigate whether the demographic variables (i.e., age, gender, racial/ethnic groups, educational level, and family income) contributed individually and collectively to the statistical prediction of mindful awareness. We found that the variables of older age, male gender, Black/African America race, and lower educational level were modestly associated with greater mindful awareness (i.e., age only accounted for 8.4% of the variability in mindful awareness), and remained significant predictors after accounting for cumulative adversity and autonomic reactivity. It is important to note that these findings are unique, as prior research found no association between mindful awareness and age (Kumar et al., 2021). Our results are also inconsistent with other research (Gan et al., 2023) which found that older individuals and males scored lower on the observing domain of mindfulness, which most closely relates to mindful awareness. Although it is encouraging to find that minority status was associated with the positive outcome of greater mindful awareness, we must interpret these results with caution, as they may have been impacted by reporting styles, cultural factors, or potential sample bias.

### 4.2 Strengths and limitations

Although the current study included a large sample size (*N* = 1,542) and produced many unique contributions to the field, it is not without limitations. The current study uses a convenience sample, which may not be representative of the greater population. To address concerns about the quality of the data collected via online self-report measures, the data underwent quality analysis through automated checks and a manual inspection. Participant responses that failed our quality checks or were completed too quickly were excluded.

While the participants varied with regard to their age and family household income, the majority identified as female (65.1%) and Caucasian (74.9%). Statistical analyses, which addressed this limitation by controlling for the demographic factors in the regression analyses, led to findings that are inconsistent with prior research. It may be that the higher levels of mindful awareness found in individuals who reported lower levels of education and identified as male gender and/or Black/African American race relates to sampling variability and unmeasured confounding factors. It is also important to consider that these demographic factors might have influenced the self-report of not only mindful awareness, but also adversity history and current functioning. Thus, future research should utilize a mixed methods approach to further explore the potential impact of demographic factors.

There are limitations related to our sole reliance on self-report measures, including the participants’ potential unwillingness or inability to fully recall adversity history. Despite this, our results are consistent with prior research (Dale et al., 2009) suggesting that individuals who report an adversity history are more negatively impacted than those who do not.

In addition, our study used the Body Perception Questionnaire (Kolacz et al., 2018; Porges, 1993), which has been found to be a valid self-report measure of autonomic reactivity. Specifically, higher scores of autonomic reactivity have been linked with data obtained from physiological measures including higher resting heart rate, lower parasympathetic activity, and less parasympathetic/sympathetic flexibility in response to challenges (Kolacz et al., 2023). Future research should use both self-report and physiological measures of heart rate variability to further assess the association between these measures and whether they differ in their relationship with cumulative adversity, mindful awareness, and current functioning.

Given the number of constructs that we were assessing in our study, we wanted to be mindful of the burden imposed on participants, and thus opted to use the Mindful Attention Awareness Scale (Brown and Ryan, 2003; Carlson and Brown, 2005), which only includes 15 items. We recognize that it would have been beneficial to use a measure, such as the Five Facet Mindfulness Questionnaire (Baer et al., 2006), that addresses other facets of mindfulness (e.g., non-reactivity and acceptance). This would have allowed us to determine whether the other facets of mindfulness may show different associations with cumulative adversity, autonomic reactivity, and current functioning in more demographically and clinically diverse samples. Alternatively, this study may have benefited from the use of the Multidimensional Assessment of Interoceptive Awareness (Mehling et al., 2012), as this measure focuses on interoception, which is an important skill that may be negatively impacted by prior adversity as suggested in polyvagal theory.

Given the cross-sectional nature of the current study, we are unable to determine temporal sequencing. Thus, the proposed mediation models serve as a theoretical model, rather than as a true causal pathway. Future studies should examine the relationship between mindful awareness, cumulative adversity, autonomic reactivity, and current functioning using a longitudinal design to address temporal precedence.

### 4.3 Conclusion and clinical implications

Our finding suggest that autonomic reactivity may mediate the relationship between cumulative adversity and mindful awareness, which in turn was associated with decreased dysfunctional coping strategies, and symptoms of emotional distress and PTSD. The finding illustrating the potential mediating role of autonomic reactivity supports the belief that prior adversity may retune the autonomic nervous system to be more reactive to perceived threat, which may in turn hinder the ability to access mindful awareness, an aspect of mindfulness important for wellbeing. Thus, our research concurs with the belief (Van der Kolk, 2006) that we need to integrate somatic, mindfulness-based approaches to address autonomic reactivity associated with prior adversity. These approaches may be useful because they may promote interoception, as this bodily awareness may lead to significant improvements in symptoms and emotional regulation, emphasizing the importance of integrating somatic approaches with mindfulness-based techniques (Neukirch et al., 2018; Payne et al., 2015; Reinhardt et al., 2020). This greater body awareness may enhance autonomic feedback and functionally improve autonomic regulation and adaptive flexibility, and may disrupt restrictive bodily patterns through movement and improve self-regulation (Van Vleet Goelz, 2025). The findings from the current study highlight the need to provide interventions that address both mindful awareness and autonomic dysregulation, especially in populations with a history of adversity (Cohen et al., 1998; Kolacz et al., 2025).

## Data Availability

The raw data supporting the conclusions of this article will be made available by the authors, without undue reservation.
